# Supplementary material for: Psychometric properties of The Baruth Protective Factors Inventory among nursing students
Source: PLoS One. 2020 Jun 5;15(6):e0233760. doi: 10.1371/journal.pone.0233760 (PMC7274377; doi:10.1371/journal.pone.0233760)
Supplement: Attachment — Submitted filename: PONE-D-19-24033_reviewer.pdf [file pone.0233760.s002.pdf]

PLOS ONE

Psychometric properties of The Baruth Protective Factors Inventory among nursing students

--Manuscript Draft--

**Manuscript Number:**	PONE-D-19-24033
**Article Type:**	Research Article
**Full Title:**	Psychometric properties of The Baruth Protective Factors Inventory among nursing students
**Short Title:**	Psychometric the protective factors inventory
**Corresponding Author:**	Hamid Sharif Nia, Ph.D<br>Mazandaran University of Medical Sciences<br>Amol, IRAN, ISLAMIC REPUBLIC OF
**Keywords:**	Resilience, Nursing, Student, Instrument development, psychometric
**Abstract:**	The Baruth Protective Factors Inventory is an instrument which assesses the risk and protective factors behind resilience. However, there is no valid or reliable Persian instrument for this resilience instrument among nursing students in Iran . This study aimed to evaluate the psychometric properties of the Persian version of The Baruth Protective Factors Inventory among nursing students. This methodological study was done in 2017. The participants were 200 nursing students who were randomly recruited from Neyshabur city. Construct validity was assessed using exploratory and confirmatory factor analysis. Reliability was also assessed through the internal consistency assessment method.<br>Exploratory factor analysis indicated a three-factor structure for the Inventory which accounted for 48.21% of its total variance. Confirmatory factor analysis confirmed the first order model. The internal consistency values of the Inventory and its domains were good, confirming its great internal consistency and reliability. The Persian version of The Baruth Protective Factors Inventory was found to have acceptable validity and reliability in order to assess resilience amongst nursing students. Therefore, the Persian version of The Baruth Protective Factors Inventory can help nursing authorities identify non-resilient students, promote their resilience, and thereby improve their academic and clinical performance. Nursing students' improved performance can positively affect both care quality and patient outcomes.
**Order of Authors:**	Tayyebeh Ali-Abadi
	Siavash Talepasand
	Christopher Boyle
	Hamid Sharif Nia, Ph.D
**Additional Information:**	
**Question**	**Response**
**Financial Disclosure**<br><br>Enter a financial disclosure statement that describes the sources of funding for the work included in this submission. Review the submission guidelines for detailed requirements. View published research articles from *PLOS ONE* for specific examples.<br><br>This statement is required for submission and **will appear in the published article** if	The author(s) received no specific funding for this work

Powered by Editorial Manager® and ProduXion Manager® from Aries Systems Corporation

the submission is accepted. Please make sure it is accurate.<br><br>**Unfunded studies**<br>Enter: *The author(s) received no specific funding for this work.*<br><br>**Funded studies**<br>Enter a statement with the following details:<br>• Initials of the authors who received each award<br>• Grant numbers awarded to each author<br>• The full name of each funder<br>• URL of each funder website<br>• Did the sponsors or funders play any role in the study design, data collection and analysis, decision to publish, or preparation of the manuscript?<br>• **NO** - Include this sentence at the end of your statement: *The funders had no role in study design, data collection and analysis, decision to publish, or preparation of the manuscript.*<br>• **YES** - Specify the role(s) played.<br><br>* typeset	
**Competing Interests**<br><br>Use the instructions below to enter a competing interest statement for this submission. On behalf of all authors, disclose any competing interests that could be perceived to bias this work—acknowledging all financial support and any other relevant financial or non-financial competing interests.<br><br>This statement **will appear in the published article** if the submission is accepted. Please make sure it is accurate. View published research articles from *PLOS ONE* for specific examples.	The authors have declared that no competing interests exist.

Powered by Editorial Manager® and ProduXion Manager® from Aries Systems Corporation

**NO authors have competing interests**<br><br>Enter: *The authors have declared that no competing interests exist.*<br><br>**Authors with competing interests**<br><br>Enter competing interest details beginning with this statement:<br><br>*I have read the journal's policy and the authors of this manuscript have the following competing interests: [insert competing interests here]*<br><br>* typeset	
**Ethics Statement**<br><br>Enter an ethics statement for this submission. This statement is required if the study involved:<br><br>• Human participants<br>• Human specimens or tissue<br>• Vertebrate animals or cephalopods<br>• Vertebrate embryos or tissues<br>• Field research<br><br>Write "N/A" if the submission does not require an ethics statement.<br><br>General guidance is provided below. Consult the submission guidelines for detailed instructions. **Make sure that all information entered here is included in the Methods section of the manuscript.**	Ethics Committee of Semnan University of Medical Sciences, Semnan, Iran (Approval Number: 96.524132)

Powered by Editorial Manager® and ProduXion Manager® from Aries Systems Corporation

**Format for specific study types**<br><br>**Human Subject Research (involving human participants and/or tissue)**<br>• Give the name of the institutional review board or ethics committee that approved the study<br>• Include the approval number and/or a statement indicating approval of this research<br>• Indicate the form of consent obtained (written/oral) or the reason that consent was not obtained (e.g. the data were analyzed anonymously)<br><br>**Animal Research (involving vertebrate animals, embryos or tissues)**<br>• Provide the name of the Institutional Animal Care and Use Committee (IACUC) or other relevant ethics board that reviewed the study protocol, and indicate whether they approved this research or granted a formal waiver of ethical approval<br>• Include an approval number if one was obtained<br>• If the study involved *non-human primates*, add *additional details* about animal welfare and steps taken to ameliorate suffering<br>• If anesthesia, euthanasia, or any kind of animal sacrifice is part of the study, include briefly which substances and/or methods were applied<br><br>**Field Research**<br><br>Include the following details if this study involves the collection of plant, animal, or other materials from a natural setting:<br>• Field permit number<br>• Name of the institution or relevant body that granted permission	
**Data Availability**<br><br>Authors are required to make all data underlying the findings described fully available, without restriction, and from the time of publication. PLOS allows rare exceptions to address legal and ethical concerns. See the PLOS Data Policy and FAQ for detailed information.	Yes - all data are fully available without restriction

Powered by Editorial Manager® and ProduXion Manager® from Aries Systems Corporation

A Data Availability Statement describing where the data can be found is required at submission. Your answers to this question constitute the Data Availability Statement and **will be published in the article**, if accepted.<br><br>**Important:** Stating 'data available on request from the author' is not sufficient. If your data are only available upon request, select 'No' for the first question and explain your exceptional situation in the text box.<br><br>Do the authors confirm that all data underlying the findings described in their manuscript are fully available without restriction?	
**Describe where the data may be found in full sentences. If you are copying our sample text, replace any instances of XXX with the appropriate details.**<br><br>• If the data are **held or will be held in a public repository**, include URLs, accession numbers or DOIs. If this information will only be available after acceptance, indicate this by ticking the box below. For example: *All XXX files are available from the XXX database (accession number(s) XXX, XXX.*).<br>• If the data are all contained **within the manuscript and/or Supporting Information files**, enter the following: *All relevant data are within the manuscript and its Supporting Information files.*<br>• If neither of these applies but you are able to provide **details of access elsewhere**, with or without limitations, please do so. For example:<br><br>*Data cannot be shared publicly because of [XXX]. Data are available from the XXX Institutional Data Access / Ethics Committee (contact via XXX) for researchers who meet the criteria for access to confidential data.*<br><br>*The data underlying the results presented in the study are available from (include the name of the third party*	All relevant data are within the manuscript and its Supporting Information files.

and contact information or URL).<br>• This text is appropriate if the data are owned by a third party and authors do not have permission to share the data.<br><br>* typeset	
Additional data availability information:	

manuscript

Click here to access/download;Manuscript;manuscript.docx

Psychometric properties of The Baruth Protective Factors Inventory among nursing students

Psychometric the protective factors inventory

Tayyebeh Ali-Abadi[1¶], Siavash Talepasand[2¶],Christopher Boyle[3¶],Hamid Sharif Nia[4*]

[1] Department of Nursing, Neyshabur Branch, Islamic Azad University, Neyshabur, Iran.

[2] Department of Psychology and Educational Sciences, Semnan University, Semnan, Iran.

[3]Education and Psychology, Graduate School of Education, University of Exeter, Exeter, UK.

[4] School of Nursing and Midwifery Amol, Mazandaran University of Medical Sciences, Sari, Iran.

* Corresponding author

Email: pegadis@yahoo.com

¶ These authors contributed equally to this work.

Abstract

The Baruth Protective Factors Inventory is an instrument which assesses the risk and protective factors behind resilience. However, there is no valid or reliable Persian instrument for this resilience instrument among nursing students in Iran .This study aimed to evaluate the psychometric properties of the Persian version of The Baruth Protective Factors Inventory among nursing students. This methodological study was done in 2017. The participants were 200 nursing students who were randomly recruited from Neyshabur city. Construct validity was assessed using exploratory and confirmatory factor analysis. Reliability was also assessed through the internal consistency assessment method.

Exploratory factor analysis indicated a three-factor structure for the Inventory which accounted for 48.21% of its total variance. Confirmatory factor analysis confirmed the first order model. The internal consistency values of the Inventory and its domains were good, confirming its great internal consistency and reliability. The Persian version of The Baruth Protective Factors Inventory was found to have acceptable validity and reliability in order to assess resilience amongst nursing students. Therefore, the Persian version of The Baruth Protective Factors Inventory can help nursing authorities identify non-resilient students, promote their resilience, and thereby improve their academic and clinical performance. Nursing students' improved performance can positively affect both care quality and patient outcomes.

Keywords: Resilience, Nursing, Student, Instrument development, psychometric

Introduction

Besides the challenges faced by most university students, nursing students face and need to cope with a wide range of challenges which are unique to their profession. For example, care delivery to patients with different physical and mental health problems, patients' death, and the risk of being infected by infectious diseases [1]. Moreover, they are affected by stressors such as poor relationships with their instructors, ambiguities in the aims of their clinical courses, lack of preparation, abundance of clinical practice, and dissatisfaction with their profession [2]. These stressors cause nursing students different levels of anxiety, stress, and discomfort [1], also high levels of stress can affect them psychologically and cause sleep disorders, reduction in their motivation for clinical activities, and undermine their self-confidence [2].

Resilience is an appropriate strategy for stress management[3]. Students' resilience can be promoted through providing themselves with protective factors such as a caring learning environment, learner-centered education, strong instructor-student relationship, and positive expectations [4]. Whereas teaching and learning processes focuses merely on information delivery, students do not get psychologically prepared for clinical practice and thus, may be vulnerable to occupational stressors later in their professional lives [4].

However resilience evaluation necessitates valid and reliable instruments but due to diversity in resilience definitions, different instruments have been developed for its measurement [5]. These instruments include, but are not limited to, Brief Resilience Scale [6], Connor-Davidson Resilience Scale [7], Baruth Protective Factor Inventory [8], Resilience Scale for Adults [9], and the Brief Resilience Coping Scale [10]. However, as resilience is affected by many factors including: context, time, age, and living conditions, its measurement tools also need to be context, time, and age-specific [11].

The conceptual framework of this study was based on the following definition of resilience: “an individualized process of development that occurs through the use of protective factors to successfully navigate perceived stress and adversities” [12]. In this framework, promotion of resilience and positive mental health necessitates minimizing the effects of risk factors (such as stressful events) and enhancing the effects of protective factors (such optimism, social support, and active coping). In other words, risk factors minimization and protective factors enhancement help promote the ability to cope with challenges in life [13].

One of the research instruments that have been used to measure protective factors specifically in adults is the Baruth Protective Factors Inventory (BPFI). This inventory is a valid and reliable measure of protective factors in an adult population through a review of resilience literature (resilience theories)[8]. The BPFI has been used to examine resilience in various contexts, including among students at a public university in the southwest United States[14], the families of disabled children in Portugal[15], the clinical and non-clinical group[16]. There were no consistent findings regarding the factor structure of BPFI when tested using Confirmatory Factor Analysis (CFA) across different cultures [14,15]. By considering this finding, previous validation studies in another setting, time and so on, is not warranted to remain valid in another time, culture or context. Therefore, regarding the deficiency of valid tool on nursing student setting the overall aim of this study was evaluation the psychometric properties of the Persian version of BPFI among nursing students. Identifying the optimal inventory structure maybe admire other researchers in this field to utilize this inventory, thus help to develop the study resilience in nursing student setting.

Materials and Methods

This methodological study was done in 2017 in order to evaluate the psychometric properties of the Persian BPFI. The study population consisted of all 376 bachelor's students who were studying nursing in Azad and medical science Universities in Neyshabur, Iran, in the 2017–2018 academic years. A sample of 200 students was recruited via simple random sampling. For instruments with less than forty items, samples of 200 were reported to be adequate [17]. Data collection was performed using BPFI. Baruth et al. developed BPFI in 2002 based on the existing literature on resilience [8]. As BPFI has 16 items, they are scored on a five-point Likert-type scale from 1 ("Strongly disagree") to 5 ("Strongly agree"). Items 1 to 4 are reverse scored. The total BPFI score varied between 16 and 80, with higher scores indicating more protective factors [16].

Procedure

After obtaining necessary permissions from its developers, the scale was translated from English into a Persian version based on World Health Organization protocol of the forward-backward translation technique [18]. Two English-Persian translators were requested to independently translate the BPFI. An expert panel, consisting of some of this article's authors, two professional translators, and a Persian-English bilingual speaker assessed and unified the two translations and constructed a single Persian translation of inventory. Thereafter, a Persian-English translator was asked to back-translate the Persian BPFI into English. This English version of the Inventory was sent to Persian-English bilingual speaker, for confirmation of the correctness of translations and confirming the similarity of the achieved English BPFI with its original. Then, the primary Persian BPFI was piloted on a sample of 28 nursing students in small two to three-person groups. So, face validity assessed this way that Students were asked to comment on the clarity of the items. Their comments required us to revise two items (9 and 10 respectively). Finally, 200 nursing students

completed the Persian BPFI and the collected data were used for construct validity and reliability assessment.

Construct validity

Exploratory Factor Analysis

The Construct validity of the scale was evaluated using Maximum Likelihood Exploratory Factor Analysis (MLEFA) with promax rotation. The Kaiser–Meyer–Olkin (KMO) test and the Bartlett's test of sphericity checked the appropriateness of the study sample and the factor analysis model. In the first step, the latent factors were extracted by based on Horn's Parallel Analysis[19]. The presence of a single item in the factor based on the $CV = 5.152 \div \sqrt{}(n-2)$ formula [20] was estimated to be approximately 0.4 (in the present formula, the CV is the number of extractable factors and n is the sample size of the study). According to the three indicator rule, at least three items must exist for each factor [21]. According to the three-indicator rule, there must be at least three items for each latent variable in the EFA [22]. Items with communalities less than 0.2 were excluded from the EFA [23].

Confirmatory Factor Analysis

The extracted factors were evaluated by Confirmatory Factor Analysis (CFA) based on Maximum Likelihood Method. CFA goodness of fit indexes include: the acceptable level of Parsimonious Normed Fit Index (PNFI), Parsimonious Comparative Fit Index (PCFI), Adjusted Goodness of Fit Index (AGFI) indices (> .5), Comparative of Fit Index (CFI), Incremental Fit Index (IFI) (> .9), Root Mean Square Error of Approximation (RMSEA): > .08 and Minimum Discrepancy Function by Degrees of Freedom divided (CMIN/DF) <3 good [24].

Convergent and Divergent Validity

The convergent and divergent validity of the BPFI was assessed using the average shared squared variance (ASV), the maximum shared squared variance (MSV) and the average

variance extracted (AVE). The convergent validity is established when AVE > 0.5 and divergent validity is established when both MSV < AVE and ASV < AVE[25].

Reliability

To assess the internal consistency of the Inventory, coefficients of Cronbach's alpha, Average Inter-Item Correlation (AIC) and Omega McDonald were calculated [26]. The values higher than 0.6 were considered acceptable [27]. The Construct Reliability (CR), which replaces Cronbach's alpha coefficient in structural equation modeling, was then assessed, and CR greater than 0.7 were considered acceptable [28].

Normal distribution, Outliers, and Missing data

The Univariate and Multivariate normal distribution of data was assessed by skewness (±3) and kurtosis (±7) [24]. The presence of multivariate outlier was investigated by Mahalanobis d-squared ($p < .001$) and multivariate normality by Mardia coefficient of multivariate kurtosis (< 20) [29]. Missing values calculated by Multiple Imputation technique (which were less than 3%) and were substituted with the mean scores of the corresponding items. All of the statistical procedures were calculated by SPSS- $AMOS_{25}$ and SPSS R-Menu v2.0.

Ethical considerations

Ethical approval for this study was provided by the Ethics Committee of Semnan University of Medical Sciences, Semnan, Iran (Approval Number: 96.524132). Necessary permissions were obtained from the developers of BPFI before its translation and psychometric evaluation. The study participants were informed about the study aim. Their agreement in responding to the BPFI items was considered to be their consent for participation in the study.

Results

A total of 200 nursing student participants were mainly female (47.5%) and single (73%) with an age mean of 21.48±2.86 (Table 1). After the score reversal of items 9 and 10 in the first stage of face validity, the results showed all items of the inventory to be appropriate, clear, and straightforward.

Table1. Participants' Sociodemographic characteristics

Characteristics		N (%)
Gender	Male	93 (46.5)
	Female	95 (47.5)
Semester	First	55 (27.5)
	Third	30 (15)
	Fifth	49 (24.5)
	Seventh	39 (19.5)
	Other	8 (4)
Marital status	Single	146 (73)
	Married	43 (21.5)

Construct validity

In this study the KMO was .816 indicating the sample size was adequate for factor analysis[30] and Bartlett's test of sphericity was significant (χ^2= 1283.25, df=105 ; p<.0001), indicating that the correlation matrix was appropriate for factor analysis[31] The parallel analysis showed a three-factor structure for BPFI which accounted for 48.21% of the total variance (Table 2).

Table2. Exploratory factor analysis of Persian version of the BPFI

Factor	Items	Factor loading	$h^{2\,a}$	Eigen value	% of variance
supportive environment	**Q10:** I have at least one caring person in my life, (whether in your family or not).	.93	.81	3.91	26.06
	**Q9:** I have a good relationship with at least one supportive person, (whether in your family or not).	.93	.88		
	**Q12:** I have at least one person who is interested in my life (whether in your family or not).	.80	.68		
	**Q11:** I can trust at least one person in my life, (whether in your family or not).	.80	.69		
Compensatory Experience	**Q15:** I have coped well with one or more major stressors in my life.	.74	.59	3.36	22.40
	**Q14:** I have control over many (but not all) events in my life.	.74	.56		
	**Q13:** I have been able to resolve many (but not all) of my problems by my self	.61	.30		
	**Q16:** I have been able to make "the best out of a bad situation" a number of times.	.56	.33		
	**Q6:** I am a creative, resourceful, and independent person.	.49	.33		
	**Q8:** I am competent and have high self-esteem.	.48	.36		
	**Q5:** I am optimistic and concentrate on the positives in most situations.	.48	.25		
Fewer Stressor	**Q4:** I have had more problems than positive experiences with work/school in the past 3 months.	.62	.38	1.67	11.13
	**Q2:** I have had more problems than positive experiences with my finances in the past 3 months	.55	.33		
	**Q3:** I have had more problems than positive experiences with my family/friends in the past 3 months	.54	.32		
	**Q1:** I have had more problems than positive experiences with my health status in the past 3 months.	.53	.30		

[a] **Communalities**

Next the extracted structure was assessed by CFA. The results of the Chi-square test for goodness-of-fit were first obtained as χ^2=134.420 (p<.0001), and other indices were then assessed for the fit of the model. All the indices (PCFI= .760, CMIN/DF=1.84, RMSEA=.065, IFI=.948 and CFI=.949) confirmed a good fit of the final model. For a better model, modification was done to the final factor structure of the BPFI that there were a covary between the measurement errors of items 6 and 15 (Figure 1).

Assessing the ASV, MSV, and AVE, also indicated that the BPFI had good convergent and divergent validity. In addition, internal consistency and CR of the BPFI were estimated to be desirable (Table3).

Table3. Convergent and divergent validity, internal consistency, and constructs reliability of BPFI.

Factor	α (CI 95)	AIC	Ω	CR	AVE	MSV	ASV
**Supportive Environment**	.926 (.908 to .941)	.764	.929	.929	.766	.260	.135
**Compensatory Experience**	.797 (.750 to .835)	.361	.801	.792	.439	.260	.175
**Fewer Stressor**	.657 (.572 to .728)	.323	.658	.658	.325	.091	.051

CR: Construct Reliability; AVE: Average Variance Extracted; MSV: Maximum Shared Squared Variance; ASV: Average Shared Squared Variance.

DISCUSSION

This study represents the first attempt to examine the reliability and validity of a Persian inventory of resilience; the BPFI among nursing students. The study was conducted in three main steps, namely that of: 1). translation and cultural adaptation, 2) data collection, and finally 3). Exploratory and confirmatory factor analysis . The result showed that the BPFI included three distinct and stable factors which explain 48.21 % of the total variance in the student nursing population.

However, the original BPFI has a four-factor structure including fewer stressors، adaptable personality ،supportive environment, compensating experiences. In primary EFA, three

factors were extracted. In this study the factors “Adaptable personality and compensatory experiences” considered as a unit. It is probable, the clients in the nonclinical group recalled their families’ adaptive appraisals and compensating experiences as a unit and the familial availability of these sources of competence contributed to their good adaptation[14]. Compensating experiences are defined as a family’s experiences of mastery within the context of adversity. This mastery includes feelings of positive control over uplifting experiences, while having experienced the same situations as hassles [14]. Masten (2001) posited the influence of family mastery resources as compensatory[32]. As registered nurses, all of the participants had developed compensatory and coping strategies which had enabled them to achieve their qualification and to progress their careers[33], however that Adaptive resources identified as the ability to plan and motivation to succeed in the future, behavioral and emotional autonomy, the capacity to handle stressful situations and access to supportive adults[34].

Items 5 and 7 were removed from the Persian version of the scale. It is valuable that the differences among different cultures can result in the instability of the factor structure. Studies in the last twenty years indicated that resilience is a multidimensional concept which is affected by context, time, age, gender, cultural origin, and living conditions. In line with our findings, factor analysis in an earlier study on 410 students at a public university in the south west of the United States revealed a four-factor structure for BPFI after the removal of item 3 [14]. Another study on the families of disabled children in Portugal also reported a four-factor structure for the scale after the removal of item 3 from the fewer stressors domain [15].

According to the final model of the BPFI, a relationship exists between the measurement error of item 6 (I am a creative, resourceful, and independent person), and 15 (I have coped well with one or more major stressors in my life). This relationship may fall, when the items

are not properly understood or due to the conceptual similarity of the sentences. Coping is a protective mechanism that eliminates or modifies the situation, controls the meaning of the experience and manage emotional consequences of the situation. Coping strategies generally seem to be more effective when an individual has more power or control over a situation [35]. Thus, this is the reason that both items have been put in the compensatory dimension of this study. Compensating experiences have been referred to as a sense of meaning and control over person-life [36] and as a person's experiences of mastery (feelings of positive control over uplifting experiences) within the context of adversity[14]. On the other hand Compensatory mechanisms include psychological adaptations such as self-efficacy (the confidence a person to their ability to do relevant self-care activities) while the ability to cope with different situations is promoted when a person's self-efficacy is high and when person feels in control during threatening situations[37]. Children and adults who have a less negative assessment of difficult situations or who consider themselves to be in control of certain situations in their lives maybe react less negatively to difficult situations and be better equipped to solve problems[38].

The factor "Social support" has been identified as a multidimensional factor that includes family, networks, community, school, employment and Consultant [14]. Social support depict medical students' resilience that experience adversity in their clinical setting[39]. In this inventory, support means a supportive environment aspect from other persons or family. A supportive learning environment promote the resilience of nursing students, reducing their vulnerability [40,41]. Staff support for students leads to stay student in the nursing program [39].

The factor "fewer stressors" assesses whether the person perceives more positive or negative experiences in their health status, finances, family and friends, and work or school [16]. ACHA identified stress, especially chronic stress as the number one barrier to college success

for most college students [42]. Nursing students inevitably experience stress in their academic education. Resilience is a dynamic process that decreased the effects of stress and prevents the potentially disabling effects of chronic stress [39].

The results of this study also showed that the items of the BPFI have an appropriate convergent and divergent validity in the final model. The convergent validity exists when the objects of the structure are close together and detect a large variance [28]. On the other hand, it has been stated that divergent validity exists when the factors of the considered structure or the latent extracted factors are completely separate from each other [43].

give more reasons not just statistical reason

The coefficients of Cronbach's alpha, Omega McDonald, AIC value confirmed the internal consistency of the overall inventory. The fewer stressors dimension presented a low coefficient of internal consistency. This result might be related to limitation of time (the last three months) and the items in this dimension (personality characteristics or environmental resources that lead to experience greater maladjustment) [15] that interact with other dimension related to personality characteristics and supportive environmental resources that help to preventing maladjustment [44].

Although data has collected from multiple samples (Azad university and Neyshabur university of medical science) to fairly generalize the factorial structure and construct validity of this inventory but the present study have several limitations. First, the study relied on self-reported data collected at one time point; thus, follow-up data would be valuable in assessment whether resilience results are consistent in demonstrating dispositional traits of individuals and can help health care authorities understand changes in student nursing resilience over time. Second, the current study did not measure test-retest reliability as a vital indicator of the scale reliability. Therefore, future research might consider measuring other indicators of reliability, such as test–retest among undergraduate college students

Despite the study limitations, the findings from the study indicated evidence of the BPFI reliability and construct validity among undergraduate nursing students and suggest the use of the inventory to effectively assess resilience. Further psychometric testing with other populations is warranted to enhance the BPFI usefulness.

Implications

With fourteen items in three domains and acceptable validity and reliability, the Persian version of BPFI can be used to diagnose non-resilience among nursing students. It is shorter than other resilience tools and can be completed in a shorter amount of time. Thus, it can be used for resilience assessment screening purposes and also for evaluating the effects of resilience-promotion interventions among nursing students in Iran.

Acknowledgments

We deeply appreciate the entire student participate in this study.

Conflict of interest

The authors have no actual or potential conflict of interests in relation to this work.

REFERENCES

1. Thomas LJ, Revell SH (2016) Resilience in nursing students: An integrative review. Nurse Education Today 36: 457-462.
2. Pourafzal F, Seyedfatemi N, Inanloo M, Haghani H (2013) Relationship between Perceived Stress with Resilience among Undergraduate Nursing Students. Hayat 19: 41-52. (persian).
3. Matua GA, Wal DMVd (2015) Living Under the Constant Threat of Ebola: A Phenomenological Study of Survivors and Family Caregivers During an Ebola Outbreak. Journal of Nursing Research 23: 217-224.
4. McAllister M, McKinnon J (2009) The importance of teaching and learning resilience in the health disciplines: a critical review of the literature. Nurse education today 29: 371-379.
5. Xie Y, Peng L, Zuo X, Li M (2016) The Psychometric Evaluation of the Connor-Davidson Resilience Scale Using a Chinese Military Sample. PLoS One 11: e0148843.

please edit for All references

6. Smith BW, Dalen J, Wiggins K, Tooley E, Christopher P, et al. (2008) The brief resilience scale: assessing the ability to bounce back. International journal of behavioral medicine 15: 194-200.

7. Connor KM, Davidson JR (2003) Development of a new resilience scale: The Connor‐ Davidson resilience scale (CD‐ RISC). Depression and anxiety 18: 76-82.

8. Baruth KE, Caroll JJ (2002) A formal assessment of resilience: The Baruth Protective Factors Inventory. The Journal of Individual Psychology.

9. Friborg O, Hjemdal O, Rosenvinge JH, Martinussen M (2003) A new rating scale for adult resilience: what are the central protective resources behind healthy adjustment? International journal of methods in psychiatric research 12: 65-76.

10. Sinclair VG, Wallston KA (2004) The development and psychometric evaluation of the Brief Resilient Coping Scale. Assessment 11: 94-101.

11. Singh K, Yu X-n (2010) Psychometric evaluation of the Connor-Davidson Resilience Scale (CD-RISC) in a sample of Indian students. Journal of Psychology 1: 23-30.

12. Stephens TM (2013) Nursing student resilience: a concept clarification. Nurs Forum 48: 125-133.

13. Abiola T, Udofia O (2011) Psychometric assessment of the Wagnild and Young's resilience scale in Kano, Nigeria. BMC Research Notes 4: 509.

14. Gardner DL, Huber CH, Steiner R, Vazquez LA, Savage TA (2008) The development and validation of the inventory of family protective factors: A brief assessment for family counseling. The Family Journal 16: 107-117.

15. Augusto CCVCd, Ferreira O, Araújo BR, Rodrigues VMCP, Figueiredo MdCAB (2014) Adaptation and validation of the Inventory of Family Protective Factors for the Portuguese culture. Revista latino-americana de enfermagem 22: 1001-1008.

16. Baruth KE (2004) The Baruth Protective Factors Inventory as a clinical assessment of resilience.

17. Lin SY, Tseng WT, Hsu MJ, Chiang HY, Tseng HC (2017) A psychometric evaluation of the Chinese version of the nursing home survey on patient safety culture. Journal of clinical nursing 26: 4664-4674.

18. World Health Organization (2016) Process of translation and adaptation of instruments.

19. Çokluk Ö, Koçak D (2016) Using Horn's Parallel Analysis Method in Exploratory Factor Analysis for Determining the Number of Factors. Educational Sciences: Theory and Practice 16: 537-551.

20. Fok D (2011) Development and Testing of a Low Vision Product Selection Instrument (LV-PSI): A Mixed-Methods Approach.

21. Munro BH (2005) Statistical methods for health care research: lippincott williams & wilkins.

22. Pett MA, Lackey NR, Sullivan JJ (2003) Making sense of factor analysis: The use of factor analysis for instrument development in health care research: Sage.

23. Hahs-Vaughn DL (2016) Applied multivariate statistical concepts: Routledge.

24. Pahlevan Sharif S, Sharif Nia H (2018) Structural Equation Modeling with AMOS. Tehran: Artin Teb.

25. Fornell C, Larcker DF (1981) Evaluating structural equation models with unobservable variables and measurement error. Journal of marketing research 18: 39-50.
26. Javali SB, Gudaganavar NV, Raj SM (2011) Effect of varying sample size in estimation of coefficients of internal consistency.
27. Altman DG (1990) Practical statistics for medical research: CRC press.
28. Hair J, Anderson R, Black B, Babin B (2016) Multivariate Data Analysis: Pearson Education.
29. Esposito Vinzi V, Chin WW, Henseler J, Wang H (2010) Handbook of partial least squares: Concepts, methods and applications: Heidelberg, Dordrecht, London, New York: Springer.
30. Tabachnick B, Fidell L (2001) Using Multivariate Statistics, Allyn and Bacon, Boston, MA. Using Multivariate Statistics, 4th ed Allyn and Bacon, Boston, MA: -.
31. Strickland OL (2003) Using factor analysis for validity assessment: practical considerations.
32. Masten AS (2001) Ordinary magic: Resilience processes in development. American psychologist 56: 227.
33. Major R, Tetley J (2019) Effects of dyslexia on registered nurses in practice. Nurse education in practice 35: 7-13.
34. Maginness A (2007) The development of resilience-a model.
35. Walton RL (2002) A comparison of perceived stress levels and coping styles of junior and senior students in nursing and social work programs: Citeseer.
36. Papalia DE, Olds SW, Feldman RD (2001) Human development (81h ed.). New York: McGraw Hill.
37. Robinson-Smith G, Johnston MV, Allen J (2000) Self-care self-efficacy, quality of life, and depression after stroke. Archives of physical medicine and rehabilitation 81: 460-464.
38. Vanderbilt-Adriance E, Shaw DS (2008) Conceptualizing and re-evaluating resilience across levels of risk, time, and domains of competence. Clinical child and family psychology review 11: 30.
39. Reyes AT, Andrusyszyn MA, Iwasiw C, Forchuk C, Babenko- Mould Y (2015) Nursing students' understanding and enactment of resilience: a grounded theory study. Journal of advanced nursing 71: 2622-2633.
40. Shiu AT, Chan CW, Lam P, Lee J, Kwong AN (2012) Baccalaureate nursing students' perceptions of peer assessment of individual contributions to a group project: a case study. Nurse Education Today 32: 214-218.
41. Chow KM, Tang WKF, Chan WHC, Sit WHJ, Choi KC, et al. (2018) Resilience and well-being of university nursing students in Hong Kong: a cross-sectional study. BMC medical education 18: 13.
42. Pope RL, Reynolds AL, Mueller JA (2019) Multicultural competence in student affairs: Jossey-Bass.
43. Hair J, Anderson RE, Tatham R, Black W (1995) Multivariate data analysis . Saddle River. NJ: Prentice Hall.

44. Bolton KW (2013) Development And Validation Of The Resilience Protective Factors Inventory: A Confirmatory Factor Analysis.

 Click here to access/download;Figure;fig1.tif

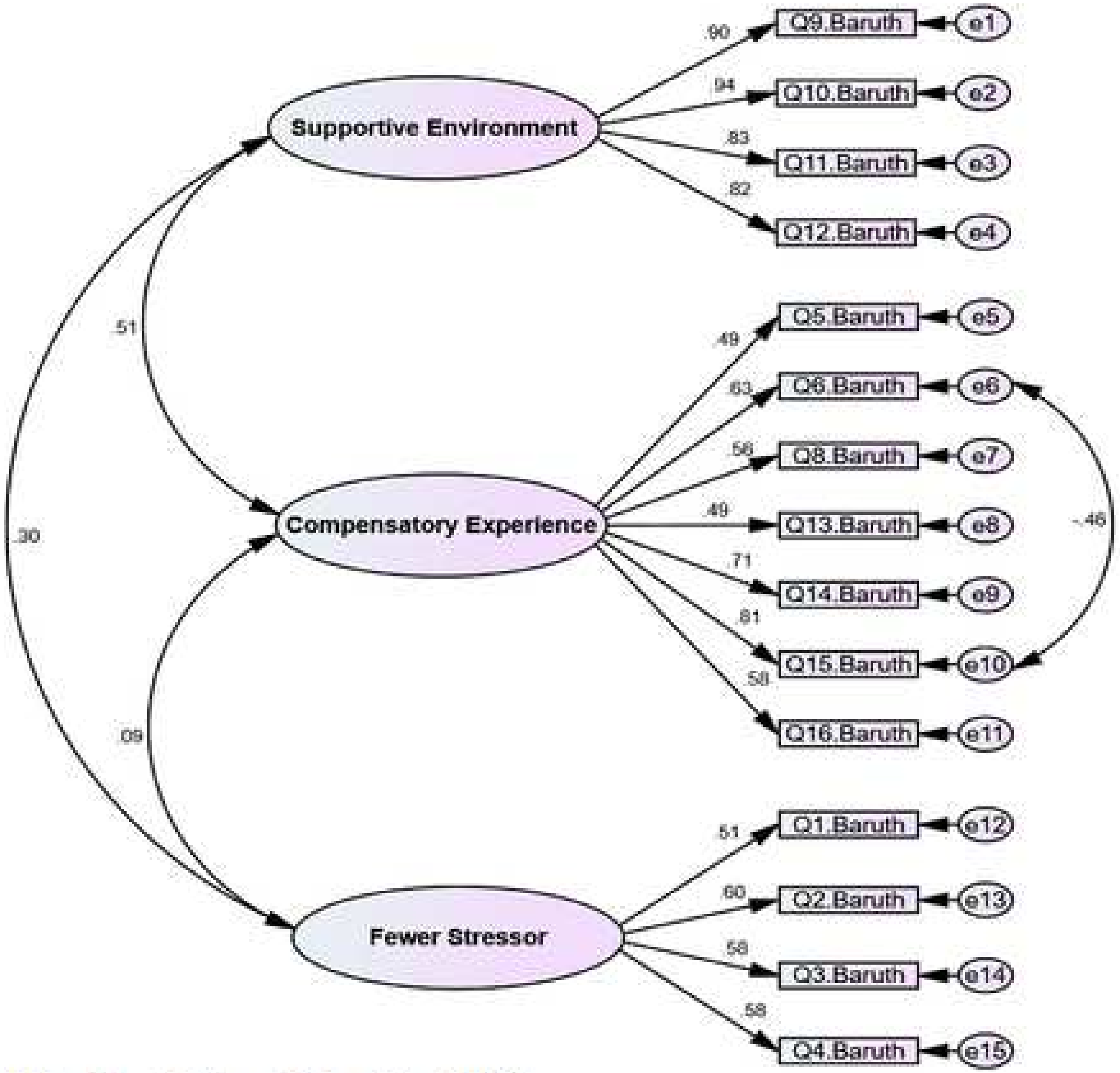

Figure 1. Final Model of the First Order CFA of the BPFI
