# Supplementary material for: Psychometric properties of The Baruth Protective Factors Inventory among nursing students
Source: PLoS One. 2020 Jun 5;15(6):e0233760. doi: 10.1371/journal.pone.0233760 (PMC7274377; doi:10.1371/journal.pone.0233760)
Supplement: Attachment — Submitted filename: Response to reviewers.doc [file pone.0233760.s003.doc]

**­­Dear Editor-in-Chief of PLOS ONE Journal**

**Dr. Joerg Heber**

Thank you for your thorough review and consideration of our manuscript “ Psychometric properties of The Baruth Protective Factors Inventory among nursing students" that was submitted to PLOS ONE Journal.

We believe that the comments and suggestions that were recommended by the reviewers have informed a much improved and more fully developed paper that will offer an important contribution to the field.

Given the word length requirements for clinical research briefs we are somewhat stymied by the extent of details we can provide However, and of critical importance, attention has been paid to developing a clearer rationale that addresses the significance of this work. More information has been provided to describe the measures used and data analysis conducted and this revised paper has more clarity about the participants of the study and discussion of culture.

We have highlighted the changes that were undertaken in response to your comments in the revised manuscript. A response to each of your comments is below.

Please feel free to contact me with any questions and concerns. I look forward to hearing from you in regards to the manuscript.

Thanks for your kind attention to the manuscript.

Sincerely, Corresponding author

**Review Comments to the Author**

| Reviewer 1 | Response |
| --- | --- |
| it would be not appropriate to use the results from EFA and CFA with the same dataset. | Thank you for your attention. This subject is explained in the limitation section.  It was highlighted in the limitation section (page 14). |
| SPSS R-Menu v2.0.” mean? | One of advantages in this study than other studies was using a Horn’s Parallel Analysis approach. This procedure is not as a command in SPSS. But there is a macro with name SPSS R-Menu v2.0 that it can be installed in SPSS. We used this macro for Horn’s Parallel Analysis.  You can find the " SPSS R-Menu v2.0" in the below link.  https://www.semanticscholar.org/paper/Determining-the-Number-of-Factors-to-Retain-in-EFA%3A-Courtney/73abb99b81129553805bd0f396cbea7e78736f97 |
| how do KMO and the Bartlett’s test of sphericity check the appropriateness of the sample and the factor analysis model | The test estimates sampling adequacy for each variable in the model and for the complete model. The statistic is a measure of the proportion of variance among variables that might be common variance. It was highlighted in the Construct validity section (page 6). |
| how to obtain the statistics for convergent and divergent validity? | In this study for estimation of convergent and divergent, we used the criterion of Fornell-Larcker (1981).  It was highlighted in the Convergent and Divergent Validity section (page 7). |
| the authors mentioned that “the results showed all items of the inventory to be appropriate, clear, and straightforward.” How did the authors make this argument? In other words, what statistical results were the authors used to make this argument? | face validity assessed clarity of the items. It was highlighted in ( page 5) |
| , What is AIC? | Average inter-item correlation (AIC) is a way of examining internal consistency reliability.  It was highlighted in the reliability section (page 7). |
| the authors mentioned that Items 5 and 7 were removed…”. I am not sure where this cam from? | The items 5 and 7 had not a good factor loading. Thus we removed them.  It was highlighted in the page 11. |

| Reviewer 2 | Response |
| --- | --- |
| Please give more reason/ analysis for the result, not just statistical explanation. Researcher can give the theoritical and contextual analysis. For example in line 276--287, according the concept/ theory and context/ student situation, why BPFI have an appropriate convergent and divergent validity in the final model? | I explain appropriateness of BPFI in nursing student setting in page3, 4 and14 and highlight it. |
