# Supplementary material for: Psychometric properties of The Baruth Protective Factors Inventory among nursing students
Source: PLoS One. 2020 Jun 5;15(6):e0233760. doi: 10.1371/journal.pone.0233760 (PMC7274377; doi:10.1371/journal.pone.0233760)
Supplement: Attachment — Submitted filename: Response to reviewers.doc [file pone.0233760.s004.doc]

**Dear Editor-in-Chief of PLOS ONE Journal**

**Dr. Joerg Heber**

**Response to minor Revision**

Thank you for your thorough review and consideration of our manuscript “ Psychometric properties of The Baruth Protective Factors Inventory among nursing students" that was submitted to PLOS ONE Journal.

We believe that the comments and suggestions that were recommended by the reviewers have informed a much improved and more fully developed paper that will offer an important contribution to the field.

Given the word length requirements for clinical research briefs, we are somewhat stymied by the extent of details we can provide, However, and of critical importance, attention has been paid to developing a clearer rationale that addresses the significance of this work. More information has been provided to describe the measures used and data analysis conducted and this revised paper has more clarity about the participants of the study and discussion of culture.

We have highlighted the changes that were undertaken in response to your comments in the revised manuscript. A response to each of your comments is below.

Please feel free to contact me with any questions and concerns. I look forward to hearing from you regarding the manuscript.

Thanks for your kind attention to the manuscript.

Sincerely, Corresponding author

**Review Comments to the Author**

| Reviewer 2 | Response |
| --- | --- |
| Please give more reason/ analysis for the result, not just statistical explanation. Researcher can give the theoretical and contextual analysis. For example in line 276--287, according to the concept/ theory and context/ student situation, why BPFI have an appropriate convergent and divergent validity in the final model? | Convergent and divergent validity in this study was evaluated by using the approach of Fornell and Larker (1981) through ASV, MSV, AVE. p: 7  The convergent validity exists when the objects of the structure are close together and detect a large variance. On the other hand, it has been stated that divergent validity exists when the factors of the considered structure or the latent extracted factors are completely separate from each other . In other words, we will not have a good convergent validity when the latent factors are not well explained by the extracted items and the items are not related to each other. For example, items 13, 14, and 16 refer to self-efficacy. Students who have higher academic self-efficacy beliefs use positive coping strategies to deal with academic difficulties. P: 14 |
| - Please edit all of references for consistently the style | Plos One reference style download and Applied. |

| Reviewer3 | Response |
| --- | --- |
| In the abstract, it is concluded that "the Persian version of The Baruth Protective Factors Inventory can help nursing authorities identify non-resilient students, promote their resilience, and thereby improve their academic and clinical performance". However, there are no research findings to support this claim. | One of the research instruments that have been used to measure protective factors specifically in adults is the Baruth Protective Factors Inventory (BPFI). A significant strong point of BPFI theory is the rich theoretical basis for it. This valid and reliable inventory measure protective factors in an adult population through a review of resilience literature (resilience theories ). Assessing resilience through BPFI increases the chance of identifying concepts and variables which are important to nursing and health care practice. Therefore this variables will be clarified and important dimensions of the variables that should be measured will be determined . Therefore, BPFI is particularly fascinating in nursing students to quickly identify stress which results in providing interventions to decrease the level of stress P: 4  Previous studies conducted during the development of the BPFI support that the instrument has content, construct, and face validity, as well as reliability in measuring the health-promotion concept of resilience in adults. p:11  As Baruth quoted Wolff (1995) identifying protective stress causing factors would play a significant role in clinical practices. Individuals with intervene risks, have further resilience while avoiding from environmental problems. When these people got further information about the reasons and protective strategies with the help of expert ones, more resilience and stability in their life would be predicable.p:4 |
| It would have been very interesting to analyze the level of stress and academic performance to see how it was associated with the validated tool. | Certainly, It would be very interesting to analyze the correlation of stress and academic performance with the validated tool. But I didn't get permission from the designers of the tools that measure stress and academic performance. |
| Furthermore, although there is a table with the descriptive variables of the sample, it does not relate them to the BPFI, when it could be interesting. Nor does it provide the descriptive data of the BPFI (mean, percentile, etc.). | Thank you for attention, but we only examined The demographic variables included age, gender, semester, and marriage. I write the descriptive data of the BPFI in the result. P:8 |
| The bibliography is not recent. Articles have been published in 2019 and 2020, for example, by the researcher Simonelli-Muñoz, which provide more current data related to the stress, academic performance and health of nursing students than those provided by the authors in this research. | Thank you for your attention to this valuable study. I cited it in my article. P: 3 |
| Reference 52 should be modified. | Plos One reference style download and Applied. |
