# Supplementary material for: Psychometric properties of The Baruth Protective Factors Inventory among nursing students
Source: PLoS One. 2020 Jun 5;15(6):e0233760. doi: 10.1371/journal.pone.0233760 (PMC7274377; doi:10.1371/journal.pone.0233760)
Supplement: Attachment — Submitted filename: Response to reviewers.doc [file pone.0233760.s005.doc]

**Dear Editor-in-Chief of PLOS ONE Journal**

**Dr. Joerg Heber**

**Response to second minor Revision**

Thank you for your thorough review and consideration of our manuscript “ Psychometric properties of The Baruth Protective Factors Inventory among nursing students" that was submitted to PLOS ONE Journal.

We believe that the comments and suggestions that were recommended by the reviewers have informed a much improved and more fully developed paper that will offer an important contribution to the field.

Given the word length requirements for clinical research briefs, we are somewhat stymied by the extent of details we can provide, However, and of critical importance, attention has been paid to developing a clearer rationale that addresses the significance of this work. More information has been provided to describe the measures used and data analysis conducted and this revised paper has more clarity about the participants of the study and discussion of culture.

We have highlighted the changes that were undertaken in response to your comments in the revised manuscript. A response to each of your comments is below.

Please feel free to contact me with any questions and concerns. I look forward to hearing from you regarding the manuscript.

Thanks for your kind attention to the manuscript.

Sincerely, Corresponding author

**Review Comments to the Author**

| Reviewer 4 | Response |
| --- | --- |
| Introduction: it argues the need to carry out this research in a well justified manner. Perhaps it is a little long. | Some sentences omitted. P:3,4 |
| Results: expressed adequately. It is not clear if in the population represented by the sample the proportion of women is also 47%. This percentage is clearly lower than in other international contexts. It is desirable to add this aspect and discuss in the discussion section whether it has any impact | There was significant difference between female and male nursing student in resilience score (p= 0.04). p:8  Gender is one of the factors influencing the understanding of psychological distress The number of male nursing students is continuing to increase.The resilience score in this study was different between male and female students, which was consistent with other studies .In these studies, the ratio between females and males were not equal but in this study, the ratio was almost the same. Also data has been collected from multiple samples (Azad university and Neyshabur university of medical science) to fairly generalize the factorial structure and construct validity of this inventory . p: 15 |
| Bibliography: it is recommended to conform to the PLoS standards. | Plos One reference style download and Applied. |
